# Exploring the Role of Gut Microbiota in Major Depressive Disorder and in Treatment Resistance to Antidepressants

**DOI:** 10.3390/biomedicines8090311

**Published:** 2020-08-27

**Authors:** Andrea Fontana, Mirko Manchia, Concetta Panebianco, Pasquale Paribello, Carlo Arzedi, Eleonora Cossu, Mario Garzilli, Maria Antonietta Montis, Andrea Mura, Claudia Pisanu, Donatella Congiu, Massimiliano Copetti, Federica Pinna, Bernardo Carpiniello, Alessio Squassina, Valerio Pazienza

**Affiliations:** 1 Unit of Biostatistics, Fondazione IRCCS Casa Sollievo della Sofferenza Hospital, 71013 San Giovanni Rotondo, Italy; a.fontana@operapadrepio.it (A.F.); m.copetti@operapadrepio.it (M.C.); 2 Unit of Psychiatry, Department of Public Health, Clinical and Molecular Medicine, University of Cagliari, 09042 Cagliari, Italy; mirkomanchia@unica.it (M.M.); pasquale.paribello@gmail.com (P.P.); carloarzedi@yahoo.it (C.A.); cossu.e90@gmail.com (E.C.); m.garzi@gmail.com (M.G.); mary.montis@tiscali.it (M.A.M.); andremura88@gmail.com (A.M.); fedepinna@inwind.it (F.P.); bcarpini@iol.it (B.C.); 3 Unit of Clinical Psychiatry, University Hospital Agency of Cagliari, 09042 Cagliari, Italy; 4 Department of Pharmacology, Dalhousie University, Halifax, NS B3H 4R2, Canada; 5 Division of Gastroenterology, Fondazione IRCCS Casa Sollievo della Sofferenza Hospital, 71013 San Giovanni Rotondo, Italy; panebianco.c@gmail.com; 6 Unit of Neuroscience and Clinical Pharmacology, Department of Biomedical Sciences, Section of Neuroscience and Clinical Pharmacology, University of Cagliari, 09042 Cagliari, Italy; claudia.pisanu@unica.it (C.P.); dcongiu@unica.it (D.C.); 7 Department of Biomedical Sciences, Division of Neuroscience and Clinical Pharmacology, University Campus, S.P. 8, Sestu-Monserrato, Km 0.700, Monserrato, 09042 Cagliari, Italy; 8 Gastroenterology Unit, Fondazione I.R.C.C.S. “Casa Sollievo della Sofferenza” Hospital, Viale dei Cappuccini 1, 71013 San Giovanni Rotondo, Italy

**Keywords:** major depressive disorder, antidepressant resistance, microbiota, gut-brain axis

## Abstract

Major depressive disorder (MDD) is a common severe psychiatric illness, exhibiting sub-optimal response to existing pharmacological treatments. Although its etiopathogenesis is still not completely understood, recent findings suggest that an altered composition of the gut microbiota might play a role. Here we aimed to explore potential differences in the composition of the gut microbiota between patients with MDD and healthy controls (HC) and to identify possible signatures of treatment response by analyzing two groups of MDD patients characterized as treatment-resistant (TR) or responders (R) to antidepressants. Stool samples were collected from 34 MDD patients (8 TR, 19 R and 7 untreated) and 20 HC. Microbiota was characterized using the 16S metagenomic approach. A penalized logistic regression analysis algorithm was applied to identify bacterial populations that best discriminate the diagnostic groups. Statistically significant differences were identified for the families of *Paenibacillaceae* and *Flavobacteriaceaea*, for the genus *Fenollaria*, and the species *Flintibacter butyricus*, *Christensenella timonensis*, and *Eisenbergiella massiliensis* among others. The phyla *Proteobacteria, Tenericutes* and the family *Peptostreptococcaceae* were more abundant in TR, whereas the phylum *Actinobacteria* was enriched in R patients. Moreover, a number of bacteria only characterized the microbiota of TR patients, and many others were only detected in R. Our results confirm that dysbiosis is a hallmark of MDD and suggest that microbiota of TR patients significantly differs from responders to antidepressants. This finding further supports the relevance of an altered composition of the gut microbiota in the etiopathogenesis of MDD, suggesting a role in response to antidepressants.

## 1. Introduction

Major depressive disorder (MDD) is a commonly occurring psychiatric condition exerting an enormous clinical and socio-economic burden [1]. The large estimated lifetime prevalence of 20% in the general population [1,2] is associated with a substantial disability burden. Indeed, MDD represented the 11th cause of global disability-adjusted life-years (DALYs) in 2017 [3]. This translates in cumbersome direct and indirect healthcare costs; MDD in 2004 was estimated to cost EUR 118 billion in Europe alone, corresponding to a cost of EUR 253 per inhabitant [4]. In this context, reducing the burden of MDD is vital. This is achievable through the application of accurate risk prediction tools, which could enable the early identification of individuals more prone to the development of MDD [5]. For instance, alexithymia, a trait characterized by the difficulty of identifying feelings and emotions and by lack of motivation, may be considered a risk factor for the development of MDD, as well as suicide attempts [6].

Similarly, treatment response prediction, if informed by reliable clinical and biological data, could increase the success of pharmacological treatment of MDD, reducing the quandary of the trial and error approach and ultimately leading to the identification of the most effective drug on an individual basis in a timely manner [7]. However, although research on risk prediction has produced clinically relevant models [8], treatment response prediction remains lacking [9]. Indeed, there have been several attempts to develop predictive algorithms of treatment resistance to antidepressants. Perlis [10] tested the performance of predictive algorithms based on a series of clinical variables drawn from the Sequenced Treatment Alternatives to Relieve Depression (STAR*D) study identifying subsets of patients at higher risk of treatment resistance with reasonable accuracy [10]. Similar predictive performances using different machine-learning approaches applied on STAR*D data were identified by Nie and coauthors [11]. Since the accuracy of predictive models informed solely by clinical data remains inadequate for clinical use, researchers have attempted to combine phenotypic and biological data to increase the performance of such models. For example, Athreya et al. added multi-omic (genomic and metabolomic) data to psychometric measures and sociodemographic factors in their model, increasing the prediction accuracy of treatment response to antidepressants from around 52% to 64% compared to a model using only clinical predictors [12]. Although other groups have proposed predictive models of TR with higher performances in accuracy and precision, [13] there is lack of replication of these results, and although encouraging, they remain distant from clinical significance.

To date, most of the studies implementing predictive models of response used information from gene sequence variants [7], while dynamic biological processes, such as modifications in the transcriptome, epigenome, proteome, metabolome or gut microbiota have been less explored. Of particular interest is the recent literature suggesting that altered gut microbiota composition might indeed interfere with the mechanism of action of antidepressants and in modulating their clinical efficacy [14]. There is extensive evidence that the microbiota is part of a bidirectional neurohumoral communication system, known as the gut–brain axis, that integrates the host gut and brain activities [15]. This system could be significantly altered in severe psychiatric disorders such as MDD, although most evidence supporting this hypothesis derives from animal models [16,17]. Some studies in humans have highlighted the presence of specific alterations of the microbiota in patients affected by MDD compared to healthy individuals. Naseribafrouei et al. found a general underrepresentation of *Bacteroidetes* in 37 patients with mild to severe MDD compared to healthy individuals [18] Jiang et al. found that in patients with MDD *Enterobacteriaceae* and *Alistipes* were over-represented, while *Faecalibacterium* were less abundant compared to healthy controls; moreover, *Faecalibacterium* were inversely correlated with symptoms severity [19]. Of interest they focused on response to antidepressants, but not on treatment resistance, finding that fecal bacterial α-diversity was increased in non-responsive but not in responsive MDD patients when compared to healthy controls [19]. Finally, Valles-Colomer et al. found that MDD corresponded to a higher prevalence of Bacteroides enterotype 2 [20]. However, little is known on the relationship between treatment resistance to antidepressants and microbiota variation. Studies have so far focused either on different phenotypes (clinical response to antidepressants) [19], or have been hampered by a limited sample size (*n* = 7 in [20]). Thus, it appears timely to investigate if and how composition of the gut microbiota might influence and correlate with treatment resistance in MDD.

In this scenario, our study had a two-fold aim. First, we explored differences in microbiota composition in patients affected by MDD compared to healthy controls (HC) (H_0_: microbiota_MDD_ = microbiota_HC_). Second, we tested whether a specific microbiota composition was associated with the presence of treatment resistance (TR) to antidepressants (H_0_: microbiota_TR_ = microbiota_R_).

## 2. Subjects and Methods

### 2.1. Participants

Thirty-four patients affected by MDD were recruited at the community mental health center of the Section of Psychiatry of the Department of Medical Science and Public Health, University of Cagliari and University Hospital Agency of Cagliari and the Unit of Clinical Pharmacology, University Hospital Agency, Cagliari, Italy. In addition, twenty healthy control (HC) subjects were recruited through word of mouth among hospital staff, their families, and university students. Assessment procedures have been detailed in Manchia et al. [21]. Briefly, patients were included in the study if: (1) they had a diagnosis of MDD according to Diagnostic and statistical manual of mental disorders DSM-IV-TR (DSM IV-TR) [22] criteria; (2) were able to express a consent to participate formulated by signing the consent form; (3) were of age between 18 and 70 years old; (4) were in euthymic phases. Patients with (1) presence of acute infections; (2) presence of chronic autoimmune inflammatory conditions (e.g., rheumatoid arthritis, thyroiditis); (3) presence of eating disorders; (4) presence of post-traumatic stress disorder; (5) presence of current substance use disorders; (6) presence of neurological disorders; (7) presence of past traumatic brain injury; (8) presence of severe co-morbidities that may influence microbiota variation were excluded. The following exclusion criteria were applied to patients with MDD and HC: (1) use of antibiotics in the three months preceding the sampling procedure, (2) chronic use of probiotics. At the time of recruitment, patients were assessed by trained mental-health professionals (psychiatry residents or senior clinical staff). We collected detailed clinical information through direct interview as well as with a systematic assessment of existing medical records. Treatment resistance for MDD was defined according to the criteria of Souery et al. [23] consisting of the presence of a poor response to at least two adequate trials of different classes of antidepressants. The assessment of TR was based on a retrospective assessment of longitudinally collected information of the clinical course as well as on evaluation of treatment response patterns. All socio-demographic and clinical information, as well as dietary, lifestyle and smoking habits, were collected at the moment of recruitment when the stool specimen was also collected. Given the bidirectional relationship between gut microbiota and drug treatment (i.e., gut microbiota could modulate treatment response and, conversely, pharmacological treatments could impact on gut microbiota), and the need to identify the specific signatures associated with TR, we analyzed three subgroups of MDD patients (treatment responsive, TR and untreated at the moment of sampling). The study protocol was approved by the Ethics Committee of the University Hospital Agency of Cagliari (PG/2018/11693) on 5 September 2018. The study was conducted in accordance with the principles of good clinical practice, with the Declaration of Helsinki and in compliance with the national legislation. Written, informed consent was obtained from all participants.

### 2.2. Sample Collection and DNA Extraction

Collection tubes with a DNA stabilization buffer (Canvax Biotech, Cordoba, Spain) were filled with fresh stool by each participant. A total of 250 µL of each sample was used to perform DNA microbial extraction using the QIAamp DNA Stool Mini Kit (Qiagen, Milan, Italy) according to the manufacturer’s protocol. After assessing DNA concentration and purity, samples were stored at −80° until processing.

### 2.3. Next-Generation Sequencing of Bacterial 16S rRNA Gene

The V3-V4 region of 16S rRNA gene was amplified using specific primers selected from Klindworth et al. [24] with Illumina adapter sequences, followed by index PCR according to the Illumina MiSeq 16S Metagenomic Sequencing Library preparation protocol, as described elsewhere [25]. Libraries were purified, quantified on a Qubit 3.0 Fluorometer (Thermo Scientific, Milan, Italy), pooled and paired-end sequenced (2 × 300 cycles) on an Illumina MiSeq (San Diego, CA, USA) platform.

### 2.4. Bioinformatic Analysis

De-multiplexed FASTQ files generated by MiSeq were analyzed using the 16S Metagenomics GAIA 2.0 software (http://www.metagenomics.cloud, Sequentia Biotech (Barcelona, Spain) 2019; Benchmark of Gaia 2.0 using published datasets available online at: http://gaia.sequentiabiotech.com/benchmark). Read pairs were quality-controlled (i.e., trimming, clipping and adapter removal) based on FastQC and BBDuk and mapped with BWA-MEM against the custom databases (based on NCBI).

### 2.5. Statistical Analysis

Clinical characteristics of patients with MDD and HC were reported as median along with interquartile range (i.e., first-third quartiles) and observed frequencies (and percentages) for continuous and categorical variables, respectively. For each continuous variable, the assumption of normality distribution was checked by means of quantile–quantile (Q–Q) plots and Shapiro–Wilks test. In the presence of non-normal distributions, comparisons between groups were performed by Mann–Whitney U test (or Kruskal–Wallis test as appropriate) and χ^2^ test (or Fisher exact test, as appropriate) for continuous and categorical variables, respectively. Stacked bar charts were used to show the gut microbiota composition (i.e., mean relative abundance %) at phylum, family, genus and species levels between MDD and HC. To identify pathways of bacterial populations that best discriminated groups (i.e., MDD versus HC or comparisons among MDD subgroups according to presence/absence of TR to antidepressants), we applied the penalized logistic regression analysis (PELORA) algorithm [26]. This algorithm is mainly used to find predictive gene signatures from microarray data by using supervised grouping techniques. To this purpose, the relative abundance (%) of each bacterium was first logistic transformed (i.e., by calculating the natural logarithm of the ratio between the relative abundance proportion and its complimentary) and then standardized (computing a Z-score) by subtracting its mean and dividing by its standard deviation (SD). Both mean and SD were computed in the sample which included all the subjects involved in the comparison. When the relative abundance was exactly 0%, the logistic transformation could not be performed for that value and, to overcome this issue, such percentage was replaced by 0.001% for the computation of Z-score only. Once a pattern was identified, its centroid was computed by the mean of the Z-scores of the involved bacteria. To calculate centroids, Z-scores of some bacteria could be sign-flipped (reversed) in order to put their values in the same direction suggested by the centroid. The PELORA algorithm was also set to accommodate clinical variables: when a new predictor is added to the model, this can either be a group centroid or a clinical variable, depending on which yields better predictive value [26]. In detail, when comparing patients with MDD versus HC, penalized logistic models which included centroid as predictor were adjusted for the effect of age at the sample collection, gender and body mass index (BMI), whereas, when comparing subgroups of patients with MDD, models were adjusted for the effect of age at MDD onset, illness duration, gender, BMI, treatment duration and the presence of concomitant drugs. Moreover, when comparing patients with MDD versus HC, covariates related to lifestyle (i.e., diet, smoke and drink habits or presence of physical activity) were not considered because they were intrinsically related to the HC profile. In accordance with the analysis protocol, two different free parameters were set in the PELORA algorithm: the number of centroids and the penalty parameter (λ). The number of centroids was set to 1, because we were mainly interested to detect only one informative pathway for each scenario, whereas a number of different combinations of λ = 0, 1/32, 1/16, 1/8, 1/4, 1/2, 1 were evaluated, performing 200 bootstrap resampling of data and recording the overall misclassification rate. For each specific scenario, the penalty parameter that achieved the lowest median misclassification rate (across the bootstrap samples) was chosen. Comparisons between Z-scores were performed using the two-sample t-test. Heatmaps of normalized Z-scores (from 0 to 1) of relative abundance of bacterial populations identified by the PELORA algorithm along with the corresponding centroid and boxplots of centroid Z-scores were produced. Two-sided *p* < 0.05 was set as the statistically significance threshold. All statistical analyses and plots were performed by the computing environment R (version 3.6, R Core Team (2020). R: A language and environment for statistical computing, Vienna, Austria), [27] (packages: supclust, ggplot2, gridExtra).

## 3. Results

### 3.1. Sample Characteristics

Since it is known that gut bacteria may influence drug metabolism, possibly affecting response to treatments and, conversely, that pharmacological treatments might alter gut microbiota composition, the study participants affected by MDD were classified in three subgroups, according to whether they had treatment-resistance to antidepressants or not or were untreated at the time of enrollment. Clinical and demographic characteristics of these three subgroups of MDD patients as well as of HC are summarized in Table 1. The four groups were homogeneous for all the examined characteristics except for gender distribution (*p* = 0.036), physical activity (*p* = 0.032), history of suicide attempt (*p* = 0.014) and intake of any concomitant drugs other than antipsychotics (*p* = 0.011). Two untreated MDD patients had a history of treatment with antipsychotics and/or mood stabilizers, but treatments were stopped a minimum of six months before the sampling procedure.

### 3.2. Comparison of Gut Microbiota Composition between Patients with MDD and HC

**Figure 1:**
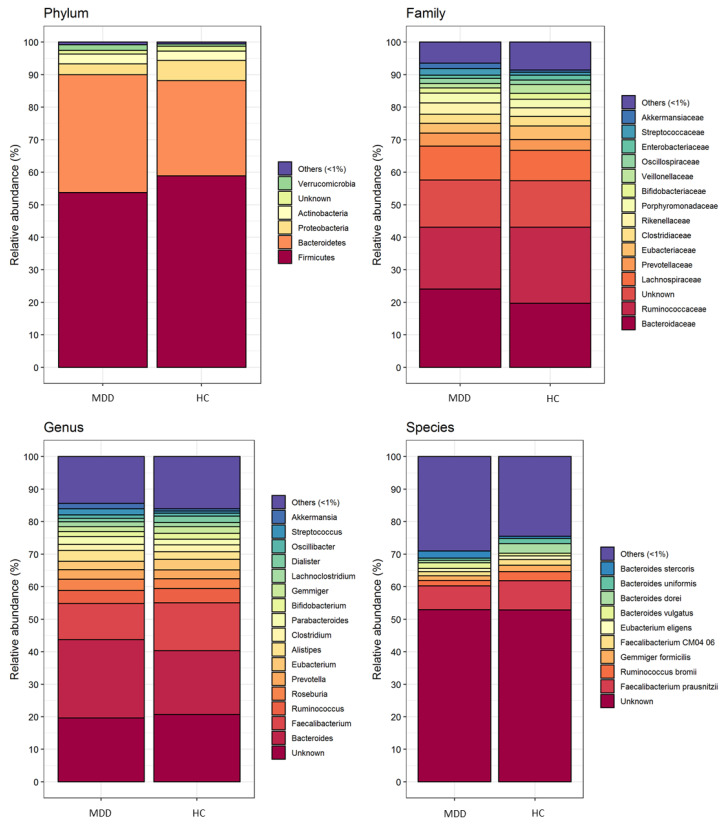
Gut microbiota composition (i.e., mean relative abundance %) at phylum, family, genus, and species levels grouped by patients with major depressive disorder (MDD) and healthy controls (HC).

In order to assess whether a different gut microbiota discriminates MDD patients and HC, its composition in the two cohorts of subjects was analyzed by next generation sequencing. A total of 8,135,346 quality-filtered read pairs were obtained from 54 study participants (34 MDD patients and 20 HC), with an average of 150,654 read pairs per sample. Gut bacterial communities at the phylum, family, genus and species level detected in MDD and HC subjects are represented in Figure 1.

**Figure 2:**
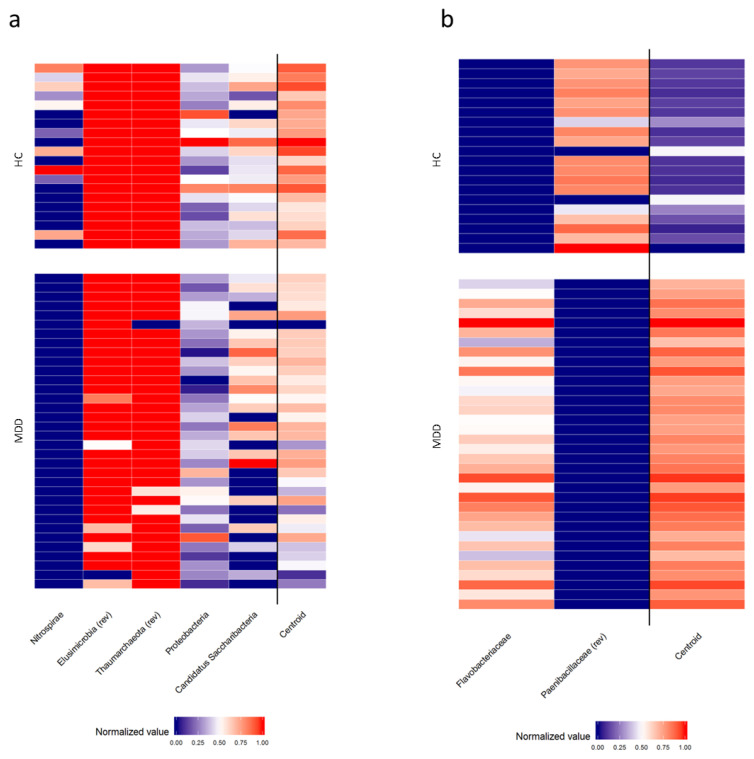

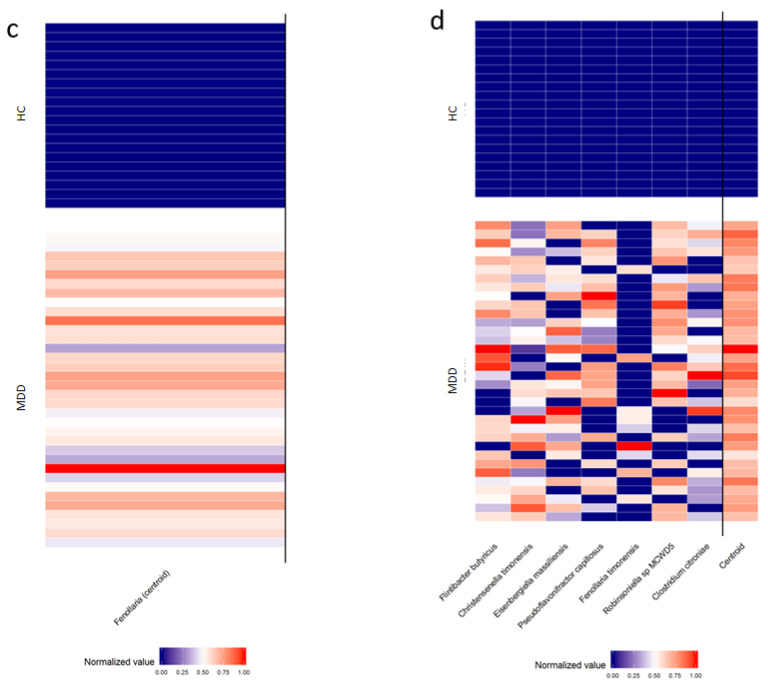
Heatmaps of relative abundance of bacterial populations (normalized Z-scores) identified by the penalized logistic regression analysis algorithm, at phylum (**a**) family (**b**), genus (**c**) and species (**d**) levels grouped by patients with major depressive disorder (MDD) and healthy controls (HC). Abbreviation (rev): the sign of the specific bacterial’s normalized value was reversed.

As expected, *Firmicutes* and *Bacteroidetes* were the most abundant phyla, accounting for about 90% of all bacteria in both groups. Based on the relative abundances generated by taxonomic analyses, the PELORA algorithm was used first to identify patterns of bacterial populations that best discriminate all patients with MDD from HC. The results are listed in Table 2 and graphically represented by the heatmap in Figure 2, showing slight variations at the phylum level, but more pronounced differences deeper in the taxonomic scale, with *Paenibacillaceae* detected in MDD but absent in HC and vice versa for the family of *Flavobacteriaceaea*, the genus *Fenollaria*, the species *Flintibacter butyricus*, *Christensenella timonensis*, *Eisenbergiella massiliensis*, *Pseudoflavonifractor capillosus*, *Fenollaria timonenis*, *Robinsoniella* sp. *MCWD5* and *Clostridum citroniae*.

**Figure 3:**
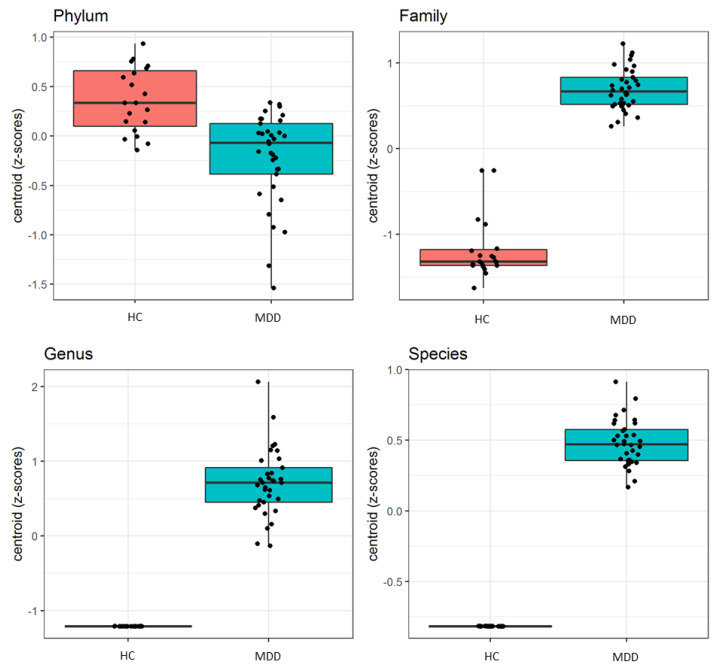
Boxplots of centroid Z-scores, computed by the penalized logistic regression analysis algorithm, which discriminated all patients with major depressive disorder (MDD) from healthy controls (HC).

In Figure 3 the distribution of centroid Z-scores computed by PELORA in MDD patients and HC shows that the identified bacterial patterns were able to discriminate between the two groups, with a higher discriminatory power at family, genus and species levels.

### 3.3. Comparison of Gut Microbiota Composition between Treated and Untreated Patients with MDD 

**Figure 4:**
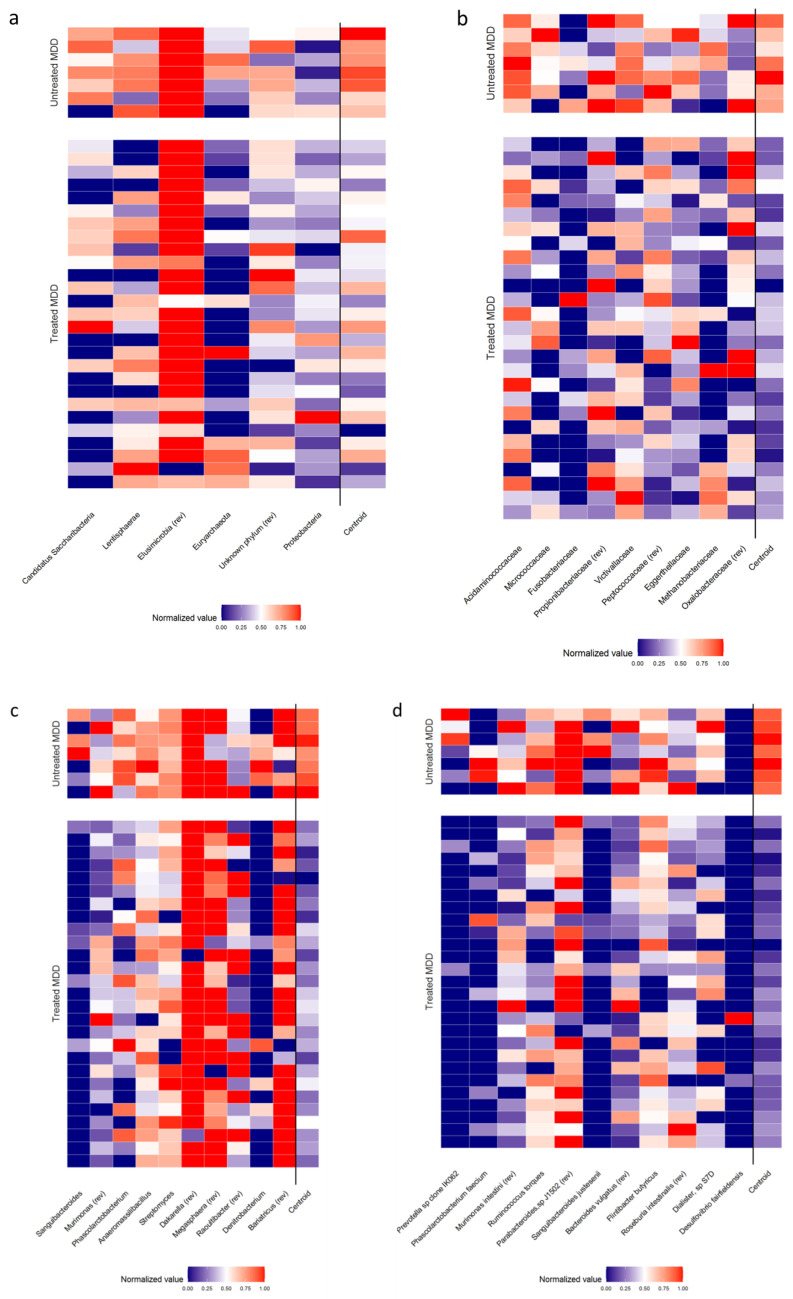
Heatmaps of relative abundance of bacterial populations (normalized Z-scores) identified the penalized logistic regression analysis algorithm, at phylum (**a**) family (**b**), genus (**c**) and species (**d**) levels grouped by treated and untreated patients with MDD. Abbreviation (rev): the sign of the specific bacterial’s normalized value was reversed.

As a second step, we investigated the eventual difference in gut microbiota in treated and untreated patients with MDD. A comparison was performed from which a conspicuous pattern of differentially represented taxa emerged, as reported in Table 3 and graphically represented by the heatmap in Figure 4. *Proteobacteria*, *Propionibacteriaceae*, *Peptococcaceae*, *Murimonas*, *Murimonas intestini*, *Parabacteroides sp J1502* were increased in treated subjects; *Candidatus Saccharibacteria*, *Lentisphaerae*, *Euryarchaeota*, *Acidaminococcaceae*, *Micrococcaceae*, *Fusibacteriaceae*, *Victivallaceae*, *Eggerthellaceae*, *Methanobacteriaceae*, *Sanguibacteroides*, *Phascolarctobacterium*, *Anaeromassilibacillus*, *Streptomyces*, *Raoultibacter*, *Denitrobacterium*, *Prevotella* sporal clone IK062, *Ruminococcus torques*, *Sanguibacteroides justesenii*, *Flintibacter butyricus*, *Roseburia intestinalis* and *Dialister sp S7D* were instead more represented in untreated patients; *Elusimirobia*, *Dakarella* and *Desulfovibrio fairfieldensis* were solely detected in the treated group.

### 3.4. Comparison of Gut Microbiota Composition between MDD Patients with and Without TR

We then evaluated the gut microbiota profile of patients classified as responders (R) and (TR). The subsequent analysis included only antidepressant-treated patients characterized for the presence/absence of TR at the time of the enrollment. As reported in Table 4, *Proteobacteria, Tenericutes* and *Peptostreptococcaceae* were more abundant in TR patients, whereas *Actinobacteria* were enriched in responders. Moreover, a number of bacteria characterized exclusively the microbiota of TR MDD patients (*Thaumarchaeota*, *Yersinia* and its species *Yersinia pseudotuberculosis*, *Peptococcus, Fenollaria timonensis*, *Blautia* sp. *canine oral taxon 337*, *Papillibacter cinnamivorans*), and many other were only detected in non-resistant patients (*Candidatus Saccharibacteria*, *Planctomycetes*, *Bacillus*, *Candidatus Soleaferrea*, *Intestinibacillus*, *Porphyromonas*, *Robinsoniella* sp. *MCWD5* and *Massilioclostridium coli*).

### 3.5. Comparison of Gut Microbiota Composition between MDD Patients with and without TR and HC

As listed in Table 5, the presence of *Elusimicrobia*, *Flavobacteriaceae*, *Fenollaria* and *Robinsoniella* sp. *MCWD5* was found exclusively in responder MDD patients, the presence of *Nitrospirae* and *Peptostreptococcaceae* only in HC and the enrichment of *Proteobacteria* in HC with respect to responders emerging as the bacterial pattern best discriminating between these two groups.

On the other hand, as reported in Table 6, the exclusive detection of *Flavobacteriaceae*, *Hungatella*, *Yersinia*, *Citrobacter*, *Fenollaria* and *Fenollaria timonensis* in patients with treatment-resistant MDD and the exclusive detection of *Candidatus Saccharibacteria* and *Massilioclostridium coli* in HC were the bacterial patterns distinguishing these groups.

## 4. Discussion

The identification of specific changes in microbiota in relation to illness status and the presence of TR to antidepressants should be interpreted in the context of several limitations. First, due to the cross-sectional design, our study was not able to establish causality (i.e., whether microbiota variation resulted from or preceded the onset of MDD). Second, the relatively small sample size could have impacted on the statistical power leading to a decreased sensitivity and specificity of our findings. Thus, we shall consider our study as hypothesis generator, capable of clarifying the strength of the association between microbiota variation and MDD and/or TR. In any instance, it should be noted that most of the available evidence in the literature has been gathered in samples of comparable size. Third, although the definition of TR was based on an accurate assessment of longitudinally collected clinical information, its assessment was mainly retrospective. However, given that TR was based on the analysis of sensible and validated psychometrics measures such as the Hamilton Depression Rating Scale [28], collected at each follow-up, it is reasonable to assume that this might have been relevant in terms of underrepresentation of TR cases, rather than of the misidentification of responsive patients as TR. Finally, although information on diet was accurately collected at the moment of stool specimen sampling, it is not possible to establish reliably how consistent were dietary habits among our patients.

However, even in light of these limitations, our study was able to identify the following relevant findings: (a) a statistically significant overrepresentation of *Paenibacillaceae* in MDD compared to HC, while the opposite was found for the family of *Flavobacteriaceaea*, the genus *Fenollaria*, the species *Flintibacter butyricus*, *Christensenella timonensis*, *Eisenbergiella massiliensis*, *Pseudoflavonifractor capillosus*, *Fenollaria timonenis*, *Robinsoniella* sp. *MCWD5* and *Clostridum citroniae*; (b) a substantial pattern of bacterial taxa was differentially represented in the comparison between untreated and treated MDD patients; (c) a number of bacteria were identifiable in the microbiota of TR MDD patients (*Thaumarchaeota*, *Yersinia* and its species *Yersinia pseudotuberculosis*, *Peptococcus*, *Fenollaria timonensis*, *Blautia* sp. *canine oral taxon 337*, *Papillibacter cinnamivorans*) but not in responsive patients; (d) compared to HC, *Flavobacteriaceae*, *Hungatella*, *Yersinia*, *Citrobacter*, *Fenollaria* and *Fenollaria timonensis* were identified exclusively in TR MDD patients, while *Elusimicrobia*, *Flavobacteriaceae*, *Fenollaria* and *Robinsoniella* sp. *MCWD5* were found exclusively in responsive MDD patients. These findings should be discussed in the context of the existing literature. There is increasing evidence of a link between gut microbiota imbalance and neurological disorders, including mood disturbances such as depression [29,30]. Supporting this link are the observations that MDD patients often exhibit metabolic and gastrointestinal symptoms [29,31] and that some antidepressants exhibit antimicrobial properties, while some antibiotics produce antidepressant effects [32].

According to the monoamine deficiency hypothesis, the main pathogenetic mechanism underlying MDD would be a shortage of monoaminergic neurotransmitters serotonin, norepinephrine or dopamine [33,34]. Consistently, the vast majority of marketed antidepressants impact the monoamine neurotransmission, with serotonin being the main target [34]. Serotonin derives from the essential amino acid tryptophan, which is introduced with diet. Once in the gut, about 90% of tryptophan enters the kynurenine pathway for the production of nicotinamide adenine dinucleotide, about 4–6% is metabolized by gut microbiota to produce indole and its derivatives, and only 3% is made available for serotonin synthesis [35,36,37]. Bacteria not only degrade tryptophan to produce indole compounds but also regulate the activity of rate-limiting enzymes in the kynurenine pathway [35,36,37], evidence that explains why gut microbiota might affect tryptophan availability for serotonin production in the brain [38].

Previous observational studies have examined the gut microbiota in patients suffering from MDD [19,39,40,41,42,43]. The novelty of our study lies in two main aspects: (1) the accurate characterization of TR cases and their numerosity leading to what is, to date, the largest sample size for microbiota analysis; and (2) the application of the PELORA algorithm to find out the most discriminative bacterial patterns at different taxonomic levels in our study population. First, we compared gut microbiota of all MDD patients with that of HC. Interestingly, we observed that all the microorganisms identified by the algorithm at the genus and species level, which were only detected in patients but not in HC, belonged to the class of *Clostridia*. Since among other gut bacteria, *Clostridia* are able to degrade tryptophan to tryptamine in the indole pathway [44], we speculate that they could divert tryptophan from serotonin production in the brain of MDD patients. Secondly, we distinguished MDD patients according to whether they were or not under pharmacological treatment at the moment of sample collection, and we observed a conspicuous number of bacteria discriminating between these two groups, thus supporting previous findings that antidepressant drugs may induce gut microbiota variations [32,45]. Some of these drug-induced changes could be potentially disadvantageous, such as the decrease in butyrate producing bacteria like *Ruminococcus torques*, *Flintibacter butyricus* and *Roseburia intestinalis* or the drop in *Phascolarctobacterium,* previously found to be correlated with positive mood [46]. Thirdly, we focused on patients with MDD who had developed TR during their illness course. Again, the relationship between antidepressants (and resistance to them) and microbiota appears to be bidirectional. If, on the one hand, antidepressant medications affect microbiota composition [32,45], on the other hand data suggest that the gut microbiota phenotype may modulate their efficacy [19,47,48]. Of note, we found that *Proteobacteria* were considerably increased in TR patients, in agreement with previous finding by Jiang et al., who observed this phylum enriched in active, non-responsive MDD patients compared with healthy subjects [19]. The phylum of *Proteobacteria* includes intestinal pathogenic microorganisms belonging to *Enterobacteriaceae,* such as *Yersinia* and *Citrobacter*, which we only found in TR patients but not in responders nor in HC. Intriguingly, previous studies reported that gastrointestinal infections caused by pathogenic bacteria, including *Citrobacter*, elicit anxiety-like behaviors [49,50,51], thereby supporting the hypothesis that these microorganisms could contribute to the failure of antidepressant trials. Striking is also the case of the genus bacterial genus *Bacillus,* which was detected in HC (data not shown), in all MDD responders, but in none of the TR MDD patients (Table 4). *Bacillus* species are able to produce norepinephrine and dopamine [52], which may contribute to restore dysregulated levels of these neurotransmitters in the brain of MDD subjects.

Overall, the results of our study confirm that gut dysbiosis is a feature of MDD patients and that the microbiota of TR subjects significantly differs from responders to antidepressants, suggesting a role of gut microbiota in the etiopathogenesis of the disease as well as in its response to therapies. Elucidating the role of microbiota in MDD pathogenesis might pave the way to a possible use of gut bacterial profiles as disease biomarkers. In addition, the identification of microorganisms discriminating treatment-resistant from responsive patients could lead to set up specific interventions of microbiota manipulation in order to improve the clinical efficacy of the current antidepressant therapies.

## Figures and Tables

**Table 1:** Demographic and clinical characteristics of patients with major depressive disorder (MDD) classified according to the presence of treatment-resistant depression (TR), responsive depression (R) and healthy controls (HC).

Variable	Level	TR MDD (*n* = 8)	R MDD (*n* = 19)	Untreated MDD (*n* = 7)	HC (*n* = 20)	*p*-Value
Age (years)	Median [IQR]	58.8 [43.7–61.6]	53.7 [43.3–59.6]	57.0 [49.1–60.9]	37.7 [30.6–58.0]	0.189 *
Gender—*n* (%)	Males	4 (50.0)	5 (26.3)	1 (14.3)	13 (65.0)	0.036 ^#^
	Females	4 (50.0)	14 (73.7)	6 (85.7)	7 (35.0)	
BMI (Kg/m^2^)	Median [IQR]	23.0 [21.9–27.8]	24.6 [21.0–28.8]	23.3 [23.1–25.2]	22.7 [21.2–23.8]	0.484 *
Family history for mental disorders—*n* (%)	No	1 (14.3)	8 (47.1)	3 (42.9)	11 (68.8)	0.117 ^#^
	Yes	6 (85.7)	9 (52.9)	4 (57.1)	5 (31.2)	
Diet—*n* (%)	Mediterranean only	5 (62.5)	10 (52.6)	4 (57.1)	18 (90.0)	0.254 ^#^
	Carbohydrates only	0 (0.0)	1 (5.3)	0 (0.0)	0 (0.0)	
	Vegetarian only	0 (0.0)	2 (10.5)	1 (14.3)	0 (0.0)	
	Mediterranean + hyperproteic	1 (12.5)	2 (10.5)	1 (14.3)	0 (0.0)	
	Mediterranean + hypercaloric	0 (0.0)	0 (0.0)	0 (0.0)	1 (5.0)	
	Mediterranean + carbohydrates	1 (12.5)	1 (5.3)	0 (0.0)	1 (5.0)	
	Mediterranean + hyperproteic + carbohydrates	0 (0.0)	1 (5.3)	1 (14.3)	0 (0.0)	
	Mediterranean + hypercaloric + carbohydrates	0 (0.0)	1 (5.3)	0 (0.0)	0 (0.0)	
	Hyperproteic + carbohydrates	1 (12.5)	0 (0.0)	0 (0.0)	0 (0.0)	
	Hypercaloric + carbohydrates + vegetarian	0 (0.0)	1 (5.3)	0 (0.0)	0 (0.0)	
Smoking habits—*n* (%)	Non-smoker	3 (37.5)	12 (63.2)	3 (42.9)	13 (65.0)	0.591 ^#^
	Smoker	4 (50.0)	3 (15.8)	2 (28.6)	4 (20.0)	
	Ex-smoker	1 (12.5)	4 (21.1)	2 (28.6)	3 (15.0)	
Drink habits—*n* (%)	None	4 (50.0)	10 (52.6)	4 (57.1)	3 (15.8)	0.086 ^#^
	One occasional drink	3 (37.5)	8 (42.1)	2 (28.6)	12 (63.2)	
	1–2 drinks per day	0 (0.0)	1 (5.3)	1 (14.3)	4 (21.1)	
	more than 1/2 L per day	1 (12.5)	0 (0.0)	0 (0.0)	0 (0.0)	
Physical activity—*n* (%)	No	6 (75.0)	11 (57.9)	5 (71.4)	5 (25.0)	0.032^#^
	Yes	2 (25.0)	8 (42.1)	2 (28.6)	15 (75.0)	
Cardiometabolic comorbidities—*n* (%)	No	6 (75.0)	14 (73.7)	6 (85.7)	16 (80.0)	0.966 ^#^
	Yes	2 (25.0)	5 (26.3)	1 (14.3)	4 (20.0)	
Age at onset (years)	Median [IQR]	25.5 [20.2–30.8]	33.0 [23.5–45.5]	40.0 [26.5–43.5]	NA	0.293 *
Disease duration (years)	Median [IQR]	26.4 [12.8–30.9]	12.7 [2.2–23.6]	17.0 [11.5–27.0]	NA	0.122 *
History of suicide attempt—*n* (%)	No	5 (62.5)	17 (94.4)	5 (71.4)	NA	0.085 ^#^
	Yes	3 (37.5)	1 (5.6)	2 (28.6)	NA	
Length of treatment with antidepressant (months)	Median [IQR]	44.0 [12.0–78.0]	24.0 [10.5–38.5]	NA	NA	0.250 *
Treatment at sample collection—*n* (%)	SSRI	4 (50.0)	8 (42.1)	NA	NA	1.000 ^#^
	SNRI/TCA/ Serotonin modulator	4 (50.0)	11 (57.9)	NA	NA	
Mood stabilizers—*n* (%)	No	5 (62.5)	17 (89.5)	5 (71.4)	NA	0.203 ^#^
	Yes	3 (37.5)	2 (10.5)	2 (28.6)	NA	
Antipsychotics—*n* (%)	No	4 (50.0)	18 (94.7)	5 (71.4)	NA	0.014 ^#^
	Yes	4 (50.0)	1 (5.3)	2 (28.6)	NA	
Any concomitant drugs—*n* (%)	No	2 (25.0)	16 (84.2)	5 (71.4)	NA	0.011 ^#^
	Yes	6 (75.0)	3 (15.8)	2 (28.6)	NA	

Missing values are excluded and only valid percentages are reported. * *p*-value from Kruskal–Wallis test; ^#^ *p*-value from Fisher exact test. Abbreviations: IQR: interquartile range (i.e., first-third quartiles); TR MDD: major depressive disorder patients with treatment-resistant depression; R MDD: responder major depressive disorder patients; untreated MDD: major depressive disorder patients that were untreated (i.e., did not receive drugs related to major depressive disorder) at the sample collection. SSRI: Selective serotonin, reuptake inhibitors. SNRI: Serotonin-norepinefrine reuptake inhibitors. TCA: Tricyclic antidepressant. NA: not applicable.

**Table 2:** Results from the penalized logistic regression analysis (PELORA) algorithm which identifies pathways of bacterial populations that best discriminate all patients with major depressive disorder (MDD) from healthy controls (HC).

Taxa Level	Bacteria Selected by PELORA	Quantity	Statistics	All MDD (*n* = 34)	HC (*n* = 20)	*p*-Value
Phylum	Proteobacteria	Relative abundance (%)	Median [IQR]	2.135 [1.591–3.461]	2.971 [2.189–4.384]	0.065 ^#^
		Z-score °	Mean ± SD	−0.192 ± 0.882	0.327 ± 1.123	
	Candidatus Saccharibacteria	Relative abundance (%)	Median [IQR]	0.005 [0.000–0.019]	0.006 [0.004–0.014]	0.132 ^#^
		Z-score °	Mean ± SD	−0.158 ± 1.128	0.268 ± 0.677	
	Nitrospirae	Relative abundance (%)	Median [IQR]	Absent	0.000 [0.000–0.006]	<0.001 ^§^
		Z-score °	Mean ± SD		0.711 ± 1.395	
	Elusimicrobia *	Relative abundance (%)	Median [IQR]	0.000 [0.000–0.000]	Absent	0.049 ^§^
		Z-score °	Mean ± SD	0.175 ± 1.233		
	Thaumarchaeota *	Relative abundance (%)	Median [IQR]	0.000 [0.000–0.000]	Absent	0.176 ^§^
		Z-score °	Mean ± SD	0.130 ± 1.249		
	Cluster centroid	Z-score (means)	Mean ± SD	−0.215 ± 0.463	0.365 ± 0.324	<0.001 ^#^
Family	Flavobacteriaceae	Relative abundance (%)	Median [IQR]	0.007 [0.004–0.015]	Absent	<0.001 ^§^
		Z-score °	Mean ± SD	0.704 ± 0.478		
	Paenibacillaceae *	Relative abundance (%)	Median [IQR]	Absent	0.011 [0.006–0.014]	<0.001 ^§^
		Z-score °	Mean ± SD		1.164 ± 0.724	
	Cluster centroid	Z-score (means)	Mean ± SD	0.694 ± 0.239	−1.180 ± 0.362	<0.001 ^#^
Genus	Fenollaria (Cluster centroid)	Relative abundance (%)	Median [IQR]	0.008 [0.004–0.012]	Absent	<0.001 ^§^
		Z-score °	Mean ± SD	0.711 ± 0.445	−1.209 ± 0.000	
Species	Flintibacter butyricus	Relative abundance (%)	Median [IQR]	0.006 [0.002–0.014]	Absent	<0.001 ^§^
		Z-score °	Mean ± SD	0.594 ± 0.790		
	Christensenella timonensis	Relative abundance (%)	Median [IQR]	0.006 [0.001–0.014]	Absent	<0.001 ^§^
		Z-score °	Mean ± SD	0.556 ± 0.864		
	Eisenbergiella massiliensis	Relative abundance (%)	Median [IQR]	0.007 [0.000–0.026]	Absent	<0.001 ^§^
		Z-score °	Mean ± SD	0.503 ± 0.949		
	Pseudoflavonifractor capillosus	Relative abundance (%)	Median [IQR]	0.006 [0.000–0.012]	Absent	<0.001 ^§^
		Z-score °	Mean ± SD	0.473 ± 0.992		
	Fenollaria timonensis	Relative abundance (%)	Median [IQR]	0.000 [0.000–0.001]	Absent	0.013 ^§^
		Z-score °	Mean ± SD	0.249 ± 1.197		
	Robinsoniella sp, MCWD5	Relative abundance (%)	Median [IQR]	0.007 [0.001–0.012]	Absent	<0.001 ^§^
		Z-score °	Mean ± SD	0.526 ± 0.914		
	Clostridium citroniae	Relative abundance (%)	Median [IQR]	0.004 [0.000–0.016]	Absent	<0.001 ^§^
		Z-score °	Mean ± SD	0.462 ± 1.007		
	Cluster centroid	Z-score (means)	Mean ± SD	0.481 ± 0.162	−0.817 ± 0.000	<0.001 ^§^

Abbreviations: IQR: interquartile range (i.e., first-third quartiles); SD: standard deviation; Absent: all values are 0%. ° Standardized Z-score: the relative abundance of each bacterium was first logistic transformed, and then the Z-score was calculated subtracting its mean and dividing by its standard deviation (SD). Both the mean and SD were computed in the sample which included all DDMs and HC. Centroid is computed by the mean of Z-scores; * to calculate the centroid, the sign of the specific bacterium’s Z-score was reversed; ^#^ *p*-values from two-sample *t*-test on Z-scores; ^§^ *p*-values from Mann–Whitney U test, calculated in the presence of no variance in one of the two groups.

**Table 3:** Results from the penalized logistic regression analysis (PELORA) algorithm which identifies pathways of bacterial populations that best discriminate all treated patients with major depressive disorder from untreated ones at sample collection.

Taxa Level	Bacteria Selected by PELORA	Quantity	Statistics	All Treated MDD (*n* = 27)	Untreated MDD (*n* = 7)	*p*-Value
Phylum	Candidatus Saccharibacteria	Relative abundance (%)	Median [IQR]	0.002 [0.000–0.016]	0.039 [0.012–0.078]	0.028 ^#^
		Z-score °	Mean ± SD	−0.189 ± 0.948	0.731 ± 0.908	
	Lentisphaerae	Relative abundance (%)	Median [IQR]	0.026 [0.001–0.090]	0.223 [0.074–0.300]	0.104 ^#^
		Z-score °	Mean ± SD	−0.142 ± 0.999	0.548 ± 0.857	
	Elusimicrobia *	Relative abundance (%)	Median [IQR]	0.000 [0.000–0.000]	Absent	0.178 ^§^
		Z-score °	Mean ± SD	0.099 ± 1.104		
	Euryarchaeota	Relative abundance (%)	Median [IQR]	0.000 [0.000–0.010]	0.004 [0.001–0.031]	0.224 ^#^
		Z-score °	Mean ± SD	−0.107 ± 1.012	0.414 ± 0.898	
	Unknown phylum *	Relative abundance (%)	Median [IQR]	1.098 [0.927–1.496]	0.842 [0.705–0.992]	0.239 ^#^
		Z-score °	Mean ± SD	0.104 ± 1.018	−0.402 ± 0.876	
	Proteobacteria	Relative abundance (%)	Median [IQR]	2.153 [1.601–3.434]	2.118 [1.181–3.516]	0.518 ^#^
		Z-score °	Mean ± SD	0.058 ± 0.998	−0.222 ± 1.054	
	Cluster centroid	Z-score (means)	Mean ± SD	−0.097 ± 0.283	0.376 ± 0.200	<0.001 ^#^
Family	Acidaminococcaceae	Relative abundance (%)	Median [IQR]	0.016 [0.004–1.165]	2.183 [0.747–3.229]	0.009 ^#^
		Z-score °	Mean ± SD	−0.222 ± 0.983	0.856 ± 0.484	
	Micrococcaceae	Relative abundance (%)	Median [IQR]	0.001 [0.000–0.004]	0.005 [0.004–0.011]	0.029 ^#^
		Z-score °	Mean ± SD	−0.188 ± 0.938	0.723 ± 0.958	
	Fusobacteriaceae	Relative abundance (%)	Median [IQR]	0.000 [0.000–0.002]	0.002 [0.000–0.049]	0.154 ^#^
		Z-score °	Mean ± SD	−0.125 ± 0.921	0.483 ± 1.218	
	Propionibacteriaceae *	Relative abundance (%)	Median [IQR]	0.059 [0.002–0.160]	0.002 [0.000–0.031]	0.159 ^#^
		Z-score °	Mean ± SD	0.124 ± 0.971	−0.478 ± 1.037	
	Victivallaceae	Relative abundance (%)	Median [IQR]	0.008 [0.000–0.044]	0.223 [0.074–0.243]	0.035 ^#^
		Z-score °	Mean ± SD	−0.182 ± 0.963	0.701 ± 0.869	
	Peptococcaceae *	Relative abundance (%)	Median [IQR]	0.017 [0.005–0.036]	0.004 [0.003–0.011]	0.130 ^#^
		Z-score °	Mean ± SD	0.133 ± 0.995	−0.513 ± 0.908	
	Eggerthellaceae	Relative abundance (%)	Median [IQR]	0.082 [0.052–0.134]	0.310 [0.125–0.565]	0.059 ^#^
		Z-score °	Mean ± SD	−0.164 ± 0.908	0.634 ± 1.156	
	Methanobacteriaceae	Relative abundance (%)	Median [IQR]	0.000 [0.000–0.010]	0.004 [0.001–0.031]	0.242 ^#^
		Z-score °	Mean ± SD	−0.103 ± 1.007	0.399 ± 0.932	
	Oxalobacteraceae *	Relative abundance (%)	Median [IQR]	0.002 [0.001–0.025]	0.002 [0.001–0.019]	0.760 ^#^
		Z-score °	Mean ± SD	0.027 ± 1.007	−0.105 ± 1.042	
	Cluster centroid	Z-score (means)	Mean ± SD	−0.141 ± 0.176	0.544 ± 0.191	<0.001 ^#^
Genus	Sanguibacteroides	Relative abundance (%)	Median [IQR]	0.000 [0.000–0.000]	0.001 [0.000–0.049]	<0.001 ^#^
		Z-score °	Mean ± SD	−0.294 ± 0.367	1.136 ± 1.738	
	Murimonas *	Relative abundance (%)	Median [IQR]	0.018 [0.005–0.040]	0.004 [0.001–0.012]	0.031 ^#^
		Z-score °	Mean ± SD	0.186 ± 0.888	−0.718 ± 1.153	
	Phascolarctobacterium	Relative abundance (%)	Median [IQR]	0.003 [0.001–0.551]	1.341 [0.080–2.583]	0.016 ^#^
		Z-score °	Mean ± SD	−0.205 ± 0.981	0.792 ± 0.638	
	Anaeromassilibacillus	Relative abundance (%)	Median [IQR]	0.007 [0.003–0.019]	0.031 [0.017–0.047]	0.033 ^#^
		Z-score °	Mean ± SD	−0.184 ± 0.974	0.708 ± 0.806	
	Streptomyces	Relative abundance (%)	Median [IQR]	0.006 [0.003–0.011]	0.015 [0.011–0.017]	0.158 ^#^
		Z-score °	Mean ± SD	−0.124 ± 1.085	0.479 ± 0.254	
	Dakarella *	Relative abundance (%)	Median [IQR]	0.000 [0.000–0.000]	Absent	0.465 ^§^
		Z-score °	Mean ± SD	0.063 ± 1.118		
	Megasphaera *	Relative abundance (%)	Median [IQR]	0.000 [0.000–0.001]	0.000 [0.000–0.022]	0.839 ^#^
		Z-score °	Mean ± SD	−0.018 ± 0.969	0.070 ± 1.195	
	Raoultibacter *	Relative abundance (%)	Median [IQR]	0.005 [0.000–0.027]	0.008 [0.003–0.020]	0.534 ^#^
		Z-score °	Mean ± SD	−0.055 ± 1.062	0.214 ± 0.736	
	Denitrobacterium	Relative abundance (%)	Median [IQR]	0.000 [0.000–0.000]	0.013 [0.000–0.044]	0.006 ^#^
		Z-score °	Mean ± SD	−0.231 ± 0.744	0.891 ± 1.392	
	Bariatricus *	Relative abundance (%)	Median [IQR]	0.000 [0.000–0.000]	0.000 [0.000–0.002]	0.513 ^#^
		Z-score °	Mean ± SD	−0.058 ± 0.960	0.225 ± 1.196	
	Cluster centroid	Z-score (means)	Mean ± SD	−0.116 ± 0.116	0.446 ± 0.072	<0.001 ^#^
Species	Prevotella sporal clone IK062	Relative abundance (%)	Median [IQR]	0.000 [0.000–0.000]	0.001 [0.000–0.081]	<0.001 ^#^
		Z-score °	Mean ± SD	−0.317 ± 0.292	1.224 ± 1.712	
	Phascolarctobacterium faecium	Relative abundance (%)	Median [IQR]	0.000 [0.000–0.000]	0.000 [0.000–1.514]	0.028 ^#^
		Z-score °	Mean ± SD	−0.189 ± 0.693	0.730 ± 1.624	
	Murimonas intestine *	Relative abundance (%)	Median [IQR]	0.017 [0.004–0.039]	0.004 [0.001–0.010]	0.038 ^#^
		Z-score °	Mean ± SD	0.179 ± 0.905	−0.691 ± 1.117	
	Ruminococcus torques	Relative abundance (%)	Median [IQR]	0.008 [0.001–0.090]	0.157 [0.077–1.008]	0.053 ^#^
		Z-score °	Mean ± SD	−0.168 ± 0.947	0.648 ± 0.998	
	Parabacteroides, sp J1502 *	Relative abundance (%)	Median [IQR]	0.001 [0.000–0.004]	0.000 [0.000–0.000]	0.088 ^#^
		Z-score °	Mean ± SD	0.149 ± 1.043	−0.575 ± 0.546	
	Sanguibacteroides justesenii	Relative abundance (%)	Median [IQR]	0.000 [0.000–0.000]	0.001 [0.000–0.046]	<0.001 ^#^
		Z-score °	Mean ± SD	−0.294 ± 0.369	1.134 ± 1.738	
	Bacteroides vulgatus *	Relative abundance (%)	Median [IQR]	1.039 [0.039–3.004]	0.015 [0.001–0.559]	0.070 ^#^
		Z-score °	Mean ± SD	0.158 ± 0.901	−0.608 ± 1.198	
	Flintibacter butyricus	Relative abundance (%)	Median [IQR]	0.006 [0.002–0.010]	0.015 [0.006–0.074]	0.059 ^#^
		Z-score °	Mean ± SD	−0.164 ± 0.985	0.632 ± 0.841	
	Roseburia intestinalis *	Relative abundance (%)	Median [IQR]	0.043 [0.015–0.302]	0.051 [0.019–0.383]	0.684 ^#^
		Z-score °	Mean ± SD	0.036 ± 0.957	−0.140 ± 1.226	
	Dialister, sp S7D	Relative abundance (%)	Median [IQR]	0.001 [0.000–0.007]	0.010 [0.003–0.011]	0.090 ^#^
		Z-score °	Mean ± SD	−0.148 ± 0.968	0.572 ± 0.977	
	Desulfovibrio fairfieldensis	Relative abundance (%)	Median [IQR]	0.000 [0.000–0.000]	Absent	0.465 ^§^
		Z-score °	Mean ± SD	0.053 ± 1.120		
	Cluster centroid	Z-score (means)	Mean ± SD	−0.159 ± 0.090	0.613 ± 0.055	<0.001 ^#^

Abbreviations: IQR: interquartile range (i.e., first-third quartiles); SD: standard deviation; Absent: all values are 0%. ° Standardized Z-score: the relative abundance of each bacterium was first logistic transformed, and then the Z-score was calculated subtracting its mean and dividing by its standard deviation (SD). Both the mean and SD were computed in the sample which included all MDD treated at sample collection and untreated MDD. Centroid is computed by the mean of Z-scores; * to calculate the centroid, the sign of the specific bacterial’s Z-score was reversed; ^#^ *p*-values from two-sample *t*-test on Z-scores; ^§^ *p*-values from Mann–Whitney U test, calculated in the presence of no variance in one of the two groups.

**Table 4:** Results from the penalized logistic regression analysis (PELORA) algorithm which identifies pathways of bacterial populations that best discriminate treatment-resistant (TR) and responder (R) MDD patients.

Taxa Level	Bacteria Selected by PELORA	Quantity	Statistics	TR MDD (*n* = 19)	R MDD (*n* = 8)	*p*-Value ^#^
Phylum	Candidatus Saccharibacteria	Relative abundance (%)	Median [IQR]	0.008 [0.002–0.019]	Absent	0.001 ^§^
		Z-score °	Mean ± SD	0.428 ± 0.889		
	Thaumarchaeota *	Relative abundance (%)	Median [IQR]	Absent	0.000 [0.000–0.001]	0.026 ^§^
		Z-score °	Mean ± SD		0.614 ± 1.761	
	Proteobacteria *	Relative abundance (%)	Median [IQR]	1.906 [1.442–2.850]	3.135 [2.102–5.772]	0.019
		Z-score °	Mean ± SD	−0.285 ± 0.732	0.677 ±1.261	
	Planctomycetes	Relative abundance (%)	Median [IQR]	0.000 [0.000–0.003]	Absent	0.023 ^§^
		Z-score °	Mean ± SD	0.247 ± 1.108		
	Actinobacteria	Relative abundance (%)	Median [IQR]	2.612 [1.441–3.709]	1.105 [0.699–1.342]	0.142
		Z-score °	Mean ± SD	0.185 ± 1.048	−0.439 ± 0.759	
	Tenericutes *	Relative abundance (%)	Median [IQR]	0.003 [0.001–0.039]	0.005 [0.001–0.128]	0.639
		Z-score °	Mean ± SD	−0.060 ± 0.980	0.143 ± 1.101	
	Cluster centroid	Z-score (means)	Mean ± SD	0.244 ± 0.230	−0.580 ± 0.314	<0.001
Family	Peptostreptococcaceae (Cluster centroid)	Relative abundance (%)	Median [IQR]	0.000 [0.000–0.000]	0.008 [0.006–0.013]	<0.001
		Z-score °	Mean ± SD	−0.581 ± 0.000	1.613 ± 0.377	
Genus	Bacillus	Relative abundance (%)	Median [IQR]	0.005 [0.003–0.008]	Absent	<0.001 ^§^
		Z-score °	Mean ± SD	0.587 ± 0.464		
	Candidatus Soleaferrea	Relative abundance (%)	Median [IQR]	0.006 [0.005–0.010]	Absent	<0.001 ^§^
		Z-score °	Mean ± SD	0.598 ± 0.411		
	Intestinibacillus	Relative abundance (%)	Median [IQR]	0.003 [0.001–0.019]	Absent	0.001 ^§^
		Z-score °	Mean ± SD	0.414 ± 0.913		
	Porphyromonas	Relative abundance (%)	Median [IQR]	0.005 [0.002–0.011]	Absent	<0.001 ^§^
		Z-score °	Mean ± SD	0.505 ± 0.731		
	Yersinia *	Relative abundance (%)	Median [IQR]	Absent	0.003 [0.001–0.020]	<0.001 ^§^
		Z-score °	Mean ± SD		1.142 ± 1.263	
	Peptococcus *	Relative abundance (%)	Median [IQR]	Absent	0.004 [0.000–0.036]	<0.001 ^§^
		Z-score °	Mean ± SD		1.078 ± 1.352	
	Cluster centroid	Z-score (means)	Mean ± SD	0.507 ± 0.136	−1.203 ± 0.132	<0.001
Species	Fenollaria timonensis	Relative abundance (%)	Median [IQR]	Absent	0.007 [0.005–0.017]	<0.001 ^§^
		Z-score °	Mean ± SD		1.446 ± 0.564	
	Robinsoniella sp, MCWD5 *	Relative abundance (%)	Median [IQR]	0.009 [0.007–0.014]	Absent	<0.001 ^§^
		Z-score °	Mean ± SD	0.603 ± 0.384		
	Massilioclostridium coli *	Relative abundance (%)	Median [IQR]	0.007 [0.006–0.013]	Absent	<0.001 ^§^
		Z-score °	Mean ± SD	0.569 ± 0.541		
	Blautia sp, canine oral taxon 337	Relative abundance (%)	Median [IQR]	Absent	0.008 [0.005–0.014]	<0.001 ^§^
		Z-score °	Mean ± SD		1.464 ± 0.485	
	Papillibacter cinnamivorans	Relative abundance (%)	Median [IQR]	Absent	0.004 [0.001–0.024]	<0.001 ^§^
		Z-score °	Mean ± SD		1.247 ± 1.090	
	Yersinia pseudotuberculosis	Relative abundance (%)	Median [IQR]	Absent	0.002 [0.001–0.020]	<0.001 ^§^
		Z-score °	Mean ± SD		1.134 ± 1.275	
	Cluster centroid	Z-score (means)	Mean ± SD	−0.567 ± 0.088	1.346 ± 0.183	<0.001

Abbreviations: IQR: interquartile range (i.e., first-third quartiles); SD: standard deviation; Absent: all values are 0%. ° Standardized Z-score: the relative abundance of each bacterium was first logistic transformed, and then the Z-score was calculated subtracting its mean and dividing by its standard deviation (SD). Both the mean and SD were computed in the sample which included all patients with and w/o TR. Centroid is computed by the mean of Z-scores; * to calculate the centroid, the sign of the specific bacterial’s Z-score was reversed; ^#^ *p*-values from two-sample t-test on Z-scores; ^§^ *p*-values from Mann–Whitney U test, calculated in the presence of no variance in one of the two groups.

**Table 5:** Results from the penalized logistic regression analysis (PELORA) algorithm which identifies pathways of bacterial populations that best discriminate responsive MDD patients (R) from healthy controls (HC).

Taxa Level	Bacteria Selected by PELORA	Quantity	Statistics	R MDD (*n* = 19)	HC (*n* = 20)	*p*-Value ^#^
Phylum	Nitrospirae	(Relative abundance (%)	Median [IQR]	Absent	0.000 [0.000–0.006]	0.001 ^§^
		Z-score °	Mean ± SD		0.483 ± 1.223	
	Proteobacteria	Relative abundance (%)	Median [IQR]	1.906 [1.442–2.850]	2.971 [2.189–4.384]	0.020
		Z-score °	Mean ± SD	−0.377 ± 0.658	0.358 ± 1.146	
	Elusimicrobia *	Relative abundance (%)	Median [IQR]	0.000 [0.000–0.001]	Absent	0.007 ^§^
		Z-score °	Mean ± SD	0.374 ± 1.350		
	Cluster centroid	Z-score (means)	Mean ± SD	−0.420 ± 0.516	0.399 ± 0.438	<0.001
Family	Peptostreptococcaceae	Relative abundance (%)	Median [IQR]	Absent	0.008 [0.004–0.011]	<0.001 ^§^
		Z-score °	Mean ± SD		0.916 ± 0.435	
	Flavobacteriaceae *	Relative abundance (%)	Median [IQR]	0.008 [0.005–0.015]	Absent	<0.001 ^§^
		Z-score °	Mean ± SD	0.967 ± 0.431		
	Cluster centroid	Z-score (means)	Mean ± SD	−0.965 ± 0.216	0.917 ± 0.217	<0.001
Genus	Fenollaria (Cluster centroid)	Relative abundance (%)	Median [IQR]	0.008 [0.004–0.010]	Absent	<0.001 ^§^
		Z-score °	Mean ± SD	0.979 ± 0.373	−0.930 ± 0.000	
Species	Robinsoniella sp, MCWD5 (Cluster centroid)	Relative abundance (%)	Median [IQR]	0.009 [0.007–0.014]	Absent	<0.001 ^§^
		Z-score °	Mean ± SD	0.981 ± 0.361	−0.932 ± 0.000	

Abbreviations: IQR: interquartile range (i.e., first-third quartiles); SD: standard deviation; Absent: all values are 0%. ° Standardized Z-score: the relative abundance of each bacterium was first logistic transformed, and then the Z-score was calculated subtracting its mean and dividing by its standard deviation (SD). Both the mean and SD were computed in the sample which included all R patients and HC. Centroid is computed by the mean of Z-scores; * to calculate the centroid, the sign of the specific bacterium’s Z-score was reversed; ^#^ *p*-values from two-sample t-test on Z-scores; ^§^ *p*-values from Mann–Whitney U test, calculated in the presence of no variance in one of the two groups.

**Table 6:** Results from the penalized logistic regression analysis (PELORA) algorithm which identifies pathways of bacterial populations that best discriminate treatment-resistant (TR) DDM patients from healthy controls (HC).

Taxa Level	Bacteria Selected by PELORA	Quantity	Statistics	TR MDD (*n* = 8)	HC (*n* = 20)	*p*-Value ^#^
Phylum	Candidatus Saccharibacteria (Cluster centroid)	Relative abundance (%)	Median [IQR]	Absent	0.006 [0.004–0.014]	<0.001 ^§^
		Z-score °	Mean ± SD		0.505 ± 0.693	
Family	Flavobacteriaceae (Cluster centroid)	Relative abundance (%)	Median [IQR]	0.007 [0.003–0.019]	Absent	<0.001 ^§^
		Z-score °	Mean ± SD	1.470 ± 0.633	−0.588 ± 0.000	
Genus	Fenollaria	(Relative abundance (%)	Median [IQR]	0.007 [0.006–0.018]	Absent	<0.001 ^§^
		Z-score °	Mean ± SD	1.484 ± 0.576		
	Hungatella	(Relative abundance (%)	Median [IQR]	0.003 [0.001–0.094]	Absent	<0.001 ^§^
		Z-score °	Mean ± SD	1.260 ± 1.148		
	Yersinia	Relative abundance (%)	Median [IQR]	0.003 [0.001–0.020]	Absent	<0.001 ^§^
		Z-score °	Mean ± SD	1.177 ± 1.281		
	Citrobacter *	Relative abundance (%)	Median [IQR]	0.000 [0.000–0.002]	Absent	0.001 ^§^
		Z-score °	Mean ± SD	0.859 ± 1.636		
	Cluster centroid	Z-score (means)	Mean ± SD	0.765 ± 0.169	−0.306 ± 0.000	<0.001
Species	Massilioclostridium coli	Relative abundance (%)	Median [IQR]	Absent	0.009 [0.008–0.013]	<0.001 ^§^
		Z-score °	Mean ± SD		0.595 ± 0.342	
	Fenollaria timonensis *	Relative abundance (%)	Median [IQR]	0.007 [0.005–0.017]	Absent	<0.001 ^§^
		Z-score °	Mean ± SD	1.486 ± 0.571		
	Cluster centroid	Z-score (means)	Mean ± SD	−1.486 ± 0.286	0.595 ± 0.171	<0.001

Abbreviations: IQR: interquartile range (i.e., first-third quartiles); SD: standard deviation; Absent: all values are 0%. ° Standardized Z-score: the relative abundance of each bacterium was first logistic transformed, and then the Z-score was calculated subtracting its mean and dividing by its standard deviation (SD). Both the mean and SD were computed in the sample which included all TR patients and HC. Centroid is computed by the mean of Z-scores; * to calculate the centroid, the sign of the specific bacterium’s Z-score was reversed; ^#^ *p*-values from two-sample t-test on Z-scores; ^§^ *p*-values from Mann–Whitney U test, calculated in the presence of no variance in one of the two groups.
